# Telephone-Based Adiposity Prevention for Families with Overweight Children (T.A.F.F.-Study): One Year Outcome of a Randomized, Controlled Trial

**DOI:** 10.3390/ijerph111010327

**Published:** 2014-10-03

**Authors:** Jana Markert, Sabine Herget, David Petroff, Ruth Gausche, Andrea Grimm, Wieland Kiess, Susann Blüher

**Affiliations:** 1 Integrated Research and Treatment Center (IFB) Adiposity Diseases, University of Leipzig, 04103 Leipzig, Germany; E-Mails: jana.markert@medizin.uni-leipzig.de (J.M); sabine.herget@medizin.uni-leipzig.de (S.H.); david.petroff@zks.uni-leipzig.de (D.P.); andrea.grimm@medizin.uni-leipzig.de (A.G.); wieland.kiess@medizin.uni-leipzig.de (W.K.); 2 Clinical Trial Centre, University of Leipzig, 04107 Leipzig, Germany; 3 CrescNet gGmbH, University of Leipzig, 04103 Leipzig, Germany; E-Mail: ruth.gausche@medizin.uni-leipzig.de; 4 Department of Women and Child Health, University Hospital for Children and Adolescents, Center for Pediatric Research, 04103 Leipzig, Germany

**Keywords:** obesity prevention, childhood and adolescence, telephone counselling, randomized controlled trial, T.A.F.F. study

## Abstract

The one-year outcome of the randomized controlled T.A.F.F. (**T**elephone based **A**diposity prevention **F**or **F**amilies) study is presented. Screening of overweight (BMI-SDS > 90th centile) children 3.5–17.4 years was performed via the German CrescNet database, and candidates were randomized to an intervention group (IG) and control group (CG). The intervention consisted of computer-aided telephone counselling for one year, supported by mailed newsletters. The primary endpoint was change in BMI-SDS; secondary endpoints were eating behavior, physical activity, media consumption, quality of life. Data from 289 families (145 IG (51% females); 144 CG (50% females)) were analyzed (Full Analysis Set: FAS; Per Protocol Set: PPS). Successful intervention was defined as decrease in BMI-SDS ≥ 0.2. In the FAS, 21% of the IG was successful as compared to 16% from the CG (95% CI for this difference: (−4, 14), *p* = 0.3, mean change in BMI-SDS: −0.02 for IG *vs.* 0.02 for CG; *p* = 0.4). According to the PPS, however, the success rate was 35% in the IG compared to 19% in the CG (mean change in BMI-SDS: −0.09 for IG *vs.* 0.02 for CG; *p* = 0.03). Scores for eating patterns (*p* = 0.01), media consumption (*p* = 0.007), physical activity (*p* = 9 × 10^−9^), quality of life (*p* = 5 × 10^−8^) decreased with age, independent of group or change in BMI-SDS. We conclude that a telephone-based obesity prevention program suffers from well-known high attrition rates so that its effectiveness could only be shown in those who adhered to completion. The connection between lifestyle and weight status is not simple and requires further research to better understand.

## 1. Introduction

Childhood obesity is a worldwide health problem affecting all age groups [1]. Although there is some evidence that obesity prevalence may have levelled off in younger children [2], it continues to increase in adolescents [3]. As many obese children and adolescents already present with associated sequelae, effective and validated obesity prevention programs for youngsters are important. However, to date, randomized controlled trials managing childhood obesity are limited [1]. A meta-analysis including 37 studies and 27,946 children has shown that lifestyle programs to prevent childhood obesity may be effective, although there is a high level of heterogeneity between interventions, the overall effects in the intervention groups were rather small (mean difference in BMI of −0.15 kg/m^2^). However, there is evidence to support beneficial effects of childhood obesity prevention programs on weight status, particularly in younger children (<12 years) [4]. A potential role for Web-based weight management programs for overweight children and adolescents has also been suggested in a systematic review [5]. In addition, a telephone-based pilot program for weight maintenance in obese youngsters has shown that regular phone contacts appear to play a potential role for effective weight maintenance in childhood obesity [6]. However, the most appropriate type of Web- or telephone-based intervention as well as the evidence level and the long-term effects need to be explored.

There is an on-going debate about parameters that might influence weight loss or weight maintenance in the long term, but only a few factors have been empirically demonstrated [7]. Beneficial factors for the prevention of childhood obesity include the involvement of parents and home settings, as these approaches seem to be superior compared to school- or out-of-home-structured settings, especially for younger children [7,8]. Thus, it has been suggested that the effectiveness of future interventions may be improved by paying careful attention to which family members are targeted and how they are involved in the intervention in terms of setting goals for behavior change and providing support [8].

However, data are scarce, particularly studies targeting all age groups including adolescents. There is also continued uncertainty about how best to involve (all) family members [9].

The T.A.F.F. study (**T**elephone based **A**diposity prevention **F**or **F**amilies) was developed as a 12- month, low threshold, parent-delivered intervention program for families with overweight or obese children, which can be offered in a community setting [10]. As data on anthropometrics, eating patterns, leisure time habits and lifestyle, psychosocial and sociodemographic parameters were collected from participating children/adolescents, mothers and fathers separately at baseline and after completion of the intervention, comprehensive information from mother-father-child triads allow detailed analyses of participating families as an entity [10,11]. Follow-up data on anthropometrics, eating patterns and lifestyle are collected 12 and 24 months after completion of the study to evaluate long-term effects of the program. The aim of this paper is to present one-year results of the T.A.F.F. program, a randomized controlled obesity prevention program based on telephone counseling for families with overweight children or adolescents.

## 2. Subjects and Methods

### 2.1. Study Design

The T.A.F.F. program addresses families with overweight or obese children and adolescents (Eligibility criteria: BMI-SDS over the 90th centile according to the German reference values [12], age 4–17 years). The program was described in more detail in earlier papers [10,11]. We performed a randomized controlled study to evaluate the efficacy of the program, registered at the German Clinical Trials Register/International Clinical Trials Registry Platform (DRKS00000803). All subjects gave their informed consent for inclusion before they participated in the study. The study was conducted in accordance with the Declaration of Helsinki, and the protocol was approved by the Ethics Committee of the Faculty of Medicine of the University of Leipzig.

### 2.2. Recruitment and Study Participants

Recruitment of overweight (BMI-SDS > 90th centile) children and adolescents was performed between 2009 and 2010 via the auxological CrescNet database [13], as previously described [10,11].

Briefly, CrescNet is a German association of independent pediatricians, which currently monitors body weight and height data from more than 550,000 children from all over Germany, and >300 pediatricians participate in the computerized data collection. Data are obtained at routine examinations in the pediatric practice and transferred on a quarterly basis (bar code label or USB-stick) to the CrescNet database [10]. CrescNet has been evaluated as a screening tool for several population-based studies [3,14]. Recruitment was performed according to the following inclusion criteria: 4–17 years of age and BMI > 90th percentile [12] (last measurement within the past six months), and a list of identified children was passed on to their local pediatrician with the request to inform the family about the weight status of its child and to forward the offer for program participation [10].

The screening detected 4005 eligible children and adolescents according to inclusion criteria. The 303 children who declared their consent to participate in the study showed a mean BMI-SDS of 1.91 [10], corresponding to an already obese weight status. Thus, the aim of the T.A.F.F. study was to prevent further weight gain in targeted individuals.

Randomization to the intervention or control group was performed with a 1:1 allocation ratio and stratified according to sex and age group (4–9 years, 10–13 years, 14–17 years) using electronically generated four-bloc-random-lists. The lists were generated before the start of the trial and assignment to trial arm was performed consecutively by a member of the team who did not have contact with participants and was not involved in data analysis. Enrolment of participants was carried out by the respective prevention manager. Written informed consent was obtained by all parents or caregivers of our study.

The trial was designed to demonstrate a change of 0.1 BMI-SDS units with a standard deviation of 0.4 with a high power of 95%. Assuming a drop-out rate of 15%, we planned to enroll 982 children. However, it became apparent that low response rates would not allow us to meet these goals, and reasons for non-participation or drop-outs have been previously analyzed and reported [10,11]. An internal committee decided to accept a lower power and carry on with the trial, thus the study was conducted with a power of 80% to demonstrate a change of 0.15 BMI-SDSunits with a standard deviation of 0.4. With an assumed drop-out rate of 15%, the goal was to enroll at least 264 children (to evaluate 112 children per arm).

### 2.3. Intervention

The core of the intervention was computer-aided telephone counseling (interviews 20–30 min each) over one year by trained prevention managers according to a standardized manual, based on family therapy approaches and solution-focused systemic therapy. Each counseling interview was preceded by the release of a newsletter (14 issues) via mail or email that addresses the specific topic of the interview (medical background of obesity and associated co-morbidities (one issue), dietary habits (three issues), eating behavior (two issues) physical activity and leisure time habits (three issues), psychological support (two issues), stress management (two issues), and a summary of the intervention including additional information (one issue), as previously described [10]. The telephone-counselling, as well as the newsletters were tailored to the age of the participating child (4–9 years, 10–13 years, 14–18 years). A detailed description of the study intervention has been previously published by our group [10]. The telephone counseling addressed the parents or caregivers of the child and primarily targeted self-regulatory capacities by solution focused counseling. The intervention consisted of 14 obligatory telephone calls every three to four weeks and two optional coaching telephone sessions at the end of the intervention as well as a final evaluation of the intervention design itself [10].

### 2.4. Outcome Measures

The primary efficacy endpoint is the change in BMI-SDS after one year of intervention. Secondary endpoints include dietary intake, level of physical activity, psychosocial well-being and health-associated quality of life.

Parameters for the analyses presented here were assessed at two timepoints, t0 and t1. The first one (t0) was at randomization, and the second (t1) at the end of the intervention (intervention group, IG) or one year following randomization (control group, CG). A detailed description of rules for choosing valid parameters is provided in Supplementary File 1.

### 2.5. Primary Endpoint

The primary endpoint is the change in BMI-SDS between baseline (t0) and termination of the intervention (t1) (BMI-SDS_t1_ − BMI-SDS_t0_). Successful completion of the intervention is defined as change in BMI-SDS ≤−0.2, according to the suggestions of the Expert Panels or Medical Associations for treatment of obesity during childhood and adolescence [15]. This indicates the stabilization of body weight by normal growing of the child or adolescent and, thus represents the prevention of further weight gain.

**Figure 1:**
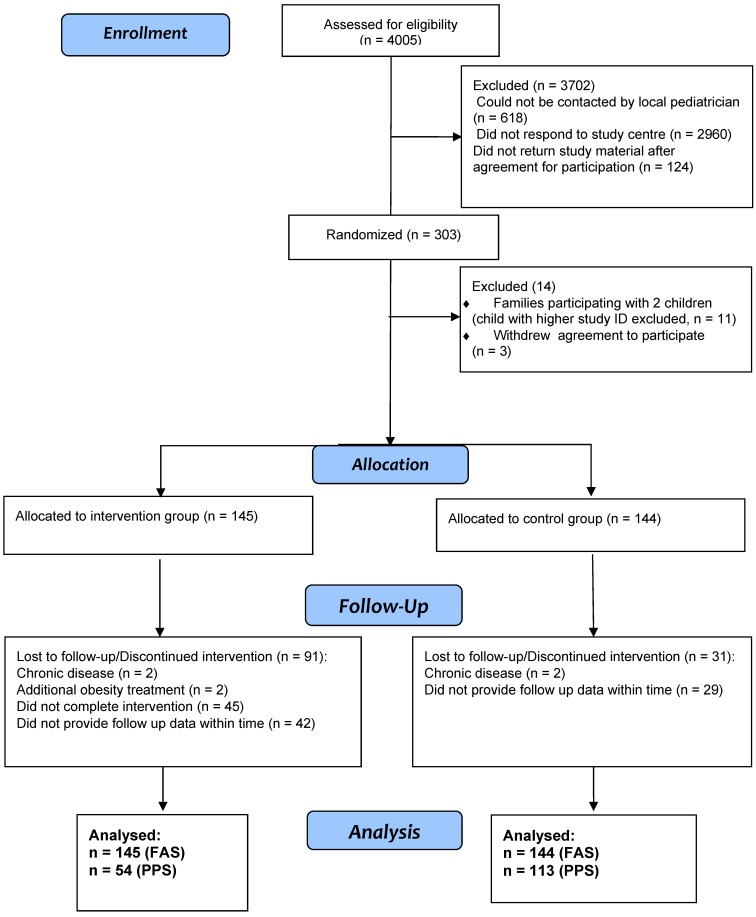
Flow chart of the study design according to the recommendations of the Consolidated Standards of Reporting Trials (CONSORT). Additional information is provided in a previous publication [11].

Two separate analyses were performed: The Full Analysis Set (FAS) consists of all randomized participants with the exception of siblings of participants already randomized to avoid bias by cluster effects and three families who withdrew agreement to participate (Figure 1). This analysis follows the “intention-to-treat” approach and considers data from all participants, including those who dropped out at any time point during the intervention. The Per Protocol Set (PPS) consists of the entire control group from the FAS that provided endpoint data and those from the intervention group who (i) finished the intervention according to the protocol (*i.e.*, had a 16th phone call), (ii) had the first anthropometric measurement no more than half a year (183 days) before receipt of the first questionnaire, and (iii) had the second anthropometric measurement within 90 days of completion of the intervention.

### 2.6. Secondary Endpoints

Secondary outcomes assessed include health-related quality of life, eating patterns, physical activity and leisure time habits (see page 10–12). All analyses of secondary endpoints were pre-specified, and no further ones were performed.

### 2.7. Anthropometric Data

Measurements of body weight and body height were assessed at 0 and 12 months of intervention by local pediatricians with standardized procedures and centrally collected in the CrescNet database as previously described [10,11]. BMI data were standardized to age and sex of the children applying German reference data [12] and were calculated as BMI-SDS. A cut off ≥1.28 SDS (90th centile) classifies overweight and a cut off ≥1.88 SDS (97th centile) classifies obesity in German children [12]. A detailed medical history was obtained from the participating child/adolescent as well the entire family in order to screen for obesity-related or underlying concomitant diseases. For the present main analysis we only refer to BMI and BMI-SDS, as the additional parameters will be subject of an upcoming data analysis.

### 2.8. Questionnaires and Scores Applied

Participants older than 10 years as well as mothers and fathers of the participants completed a questionnaire at t0 and at t1 to obtain triad-information on eating behavior, leisure time habits and lifestyle, level of daily physical activity, media consumption, psychosocial factors and quality of life. Parents of children younger than 10 years completed a proxy-report for their children. The questionnaire consisted, apart from questions regarding the intervention itself, of validated and published scales and provided information on the following obesity-related topics.

*Eating patterns*: The food frequency questionnaire used in the KIGGS study (individual eating habits) and the AD-EVA questionnaires (family eating habits) [16,17,18] were applied. An eating behavior score was calculated based on four basic areas of eating habits: number of meals per day, joint meals within the family, activity during meals, regular mealtimes. Combining these four values results in a nine point scale to run from −10 (bad) to +10 (good) in steps of 2.5. Detailed information is provided in Supplementary File 2a.

*Physical activity:* The level of physical activity was assessed, based on the questionnaires used by the German health Interview and Examination Survey for children and adolescents (KiGGS) [19] as well as the physical activity scale (MoMo-questionnaire) [20]. The information on whether a child was a member of a sports club (yes/no) as well as what was the participant’s motivation to perform physical activity was also included in the analyses [21]. A physical activity score was calculated based on the answer to two questions from the questionnaires, intending to reflect the amount of time being active both in organized sports and in one’s own free time (Supplementary File 2b).

*Media consumption:* Information on media consumption (and leisure time habits) was obtained applying the KIGGS-questionnaire [22]. A media consumption score was calculated, based on the answers to two questions and provides an estimate for the average number of minutes spent per day using these media (Supplementary File 2c). The scores resulting from both questions were added. If one was missing, then the other was treated as zero.

*Health-related quality of life*: Health-related quality of life was assessed by the KINDL-R questionnaire [23]. Additional parameters that were obtained include resources and protection factors for health-related quality of life [24], expectancy for self-efficacy [25], subjective life satisfaction [26] and information on social support [27]. For the analyses presented here, items from the KINDL-R were utilized (see Supplementary File 2d). Higher score values correspond with a higher quality of life, with a high degree of reliability and validity [23].

### 2.9. Statistical Analyses

Data are summarized using mean and standard deviation (SD) or proportions, as appropriate. Proportions are compared using a chi-squared statistic without Yate’s continuity correction, and the corresponding confidence interval is found by inverting the test. Comparisons of means alone used (paired) t-tests with Welch’s approximation. Linear models were analyzed with ANCOVA using type III sums of squares.

The high drop-out rates typical in the field of obesity programs mean that particular care must be taken in the analyses. In comparing success rates for the FAS, we make the conservative assumption that those who did not provide data at follow-up, were unsuccessful. The estimates for the changes in BMI-SDS rely on an imputation technique where the mean value of the quantity does not change, but preserving the observed variance. For secondary endpoints, analyses were performed initially with existing data and where significant changes between baseline and follow-up or any observed group effects were to be tested for robustness using similar imputation techniques.

The significance level was placed at 5%. All analyses and figures made use of R version 2.14 [28].

## 3. Results

A total of 303 families completed the study materials and started the intervention [10]. Of these, 11 families participated with two children. In order to avoid cluster effects, the child with the higher study ID was excluded from analyses. Three families withdrew their written agreement for participation thus, data from 289 families were utilized for further analyses (145 intervention group, 51% females, and 144 control group, 50% females) (Figure 1). At baseline, 92 participants (31.8%) were <8 years, 126 (43.6%) between the ages of 8 and 12 years and 71 (24.6%) who were at least 12 years of age. Detailed baseline characteristics are presented in Table 1.

**Table 1:** Baseline characteristics. Data are presented as mean ± SD or as numbers (%).

Parameter	Intervention (n = 145)	Control (n = 144)	*p*-value
Number of females	74 (51.0%)	72 (50.0%)	1.0
Age (years)	9.7 ± 3.0	9.8 ± 3.1	0.8
Height (cm)	143.3 ± 18.0	143.6 ± 18.7	0.9
Weight (kg)	51.6 ± 19.9	51.9 ± 19.0	0.9
BMI (kg/m^2^)	24.1 ± 4.2	24.2 ± 3.5	0.8
BMI-SDS	2.00 ± 0.52	2.04 ± 0.47	0.5
Eating behavior score	5.5 ± 3.4	5.4 ± 3.6	0.7
Physical activity score	18.3 ± 10.7	17.1 ± 10.2	0.4
Media score	141.2 ± 88.6	163.7 ± 114.0	0.06
KINDL score	93.8 ± 12.9	93.4 ± 11.3	0.8

### 3.1. Primary Efficacy Endpoint

**Figure 2:**
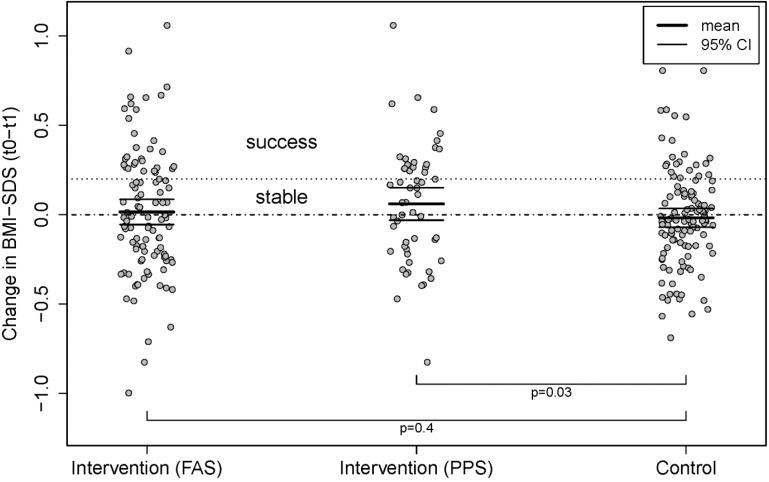
The primary endpoint (change in BMI-SDS) for the intervention group, presented as full protocol set (intention-to-treat, ITT / FAS; estimated change in BMI-SDS: −0.015 (95% CI (−0.09, 0.06)) and per protocol set (PPS; estimated change in BMI-SDS: −0.086 (95% CI (−0.18, 0.01)) compared to the control group (estimated change in BMI-SDS: 0.018 (95% CI (−0.03, 0.07)). ANCOVA analysis for the PPS revealed that the coefficient for the control group is 0.11 (95% CI (0.01, 0.20), *p* = 0.03), demonstrating that the therapy is effective for those who follow it to completion.

*Full analysis set (FAS)*: In total, 30/145 (21%) of the intervention group were successful in losing weight as compared to 23/144 (16%) from the control group (95% CI for this difference: (−4, 14), *p* = 0.3). The mean change in BMI-SDS for the intervention group was −0.015 (95% CI (−0.09, 0.06)) compared to the control group 0.018 (95% CI (−0.03, 0.07)) (Figure 2). A linear model was considered with the change in BMI-SDS as the dependent variable, the group as a factor and the time interval between anthropometric measures at t0 and t1 as well as the baseline value for BMI-SDS as covariates. A step-wise adding and dropping of terms depending on AIC showed that the time between measurements is not informative and was thus left out of the final model. There are three coefficients in the final model. The intercept is 0.24 (95% CI (0.06, 0.42), *p* = 0.009) indicating the expected overall slight increase in BMI-SDS values. The coefficients in front of the baseline BMI-SDS is −0.13 (95% CI (−0.21, −0.04), *p* = 0.003), confirming the expectation that those with higher initial values for BMI-SDS decrease it by a larger amount on average. The coefficient for the control group is 0.04 (95% CI (−0.05, 0.12), *p* = 0.4).

*Per protocol set (PPS):* Success rates in the per protocol set are 19/54 (35%) in the intervention arm and 22/113 (19%) in the control arm (95% CI for the difference: (1, 30), *p* = 0.03). The mean change in BMI-SDS for the intervention group was −0.086 (95% CI (−0.18, 0.01)) and for the control group 0.018 (95% CI (−0.03, 0.07), see FAS) (Figure 2). For the linear model, the intercept is 0.16 (95% CI (−0.03, 0.36), *p* = 0.1). The coefficient in front of the baseline BMI-SDS is −0.12 (95% CI (−0.21, −0.03), *p* = 0.007), coinciding very well for the dependence on the baseline value of BMI-SDS that was estimated from the FAS. The coefficient for the control group is 0.11 (95% CI (0.01, 0.20), *p* = 0.03), demonstrating that the therapy is effective for those who follow it according to protocol and to completion (Figure 2).

### 3.2. Secondary Endpoints

**Figure 3:**
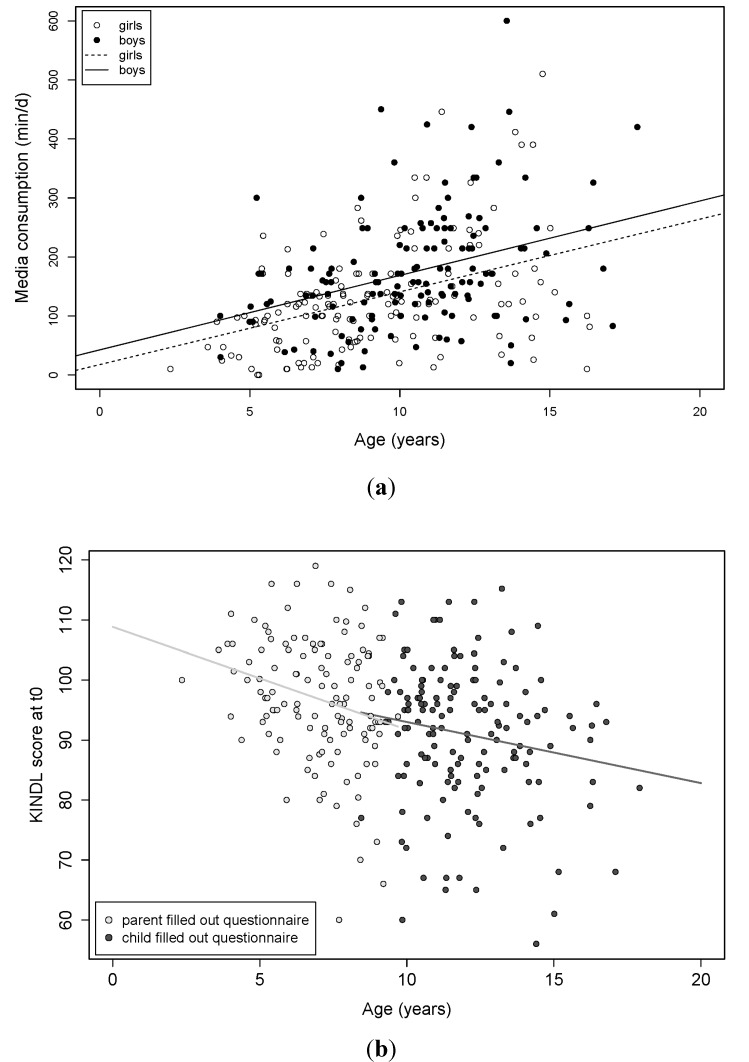
(**a**) Media scores at t0 as they depend on age. Please note that questionnaires were completed by parents for children ≤10 years and by children/adolescents themselves for participants older than 10 years (linear correlation coefficient 0.2 (95% CI (0.07, 0.4), *p* = 0.007). Differences in media consumption are also evident depending on gender: mean media score for boys (black circles, black line) is 176 as compared to 129 for girls (white circles, dashed line) (95% CI for the difference in means is (24, 70), *p* = 9 × 10^−5^). (**b**) Correlation between KINDL-R scores and age at baseline (t0). Please note that this age dependence can be found independent on who filled out the questionnaire (parents or participants themselves) (correlation coefficient: −0.32, 95% CI (−0.42, −0.21), *p* = 5 × 10^−8^).

Baseline media consumption over all age groups as well as health-related quality of life are presented in an exemplary manner in Figure 3a and Figure 3b.

*Media consumption*: Information on media consumption is available for 287 participants at t0 and for 192 participants at t1. At t0, one quarter of the participants spent less than 91 min with media, one half spent more than 134 min and one quarter more than 209 min daily. There is a significant dependence on age, starting at about 10 years (linear correlation coefficient 0.2 (95% CI (0.07, 0.4), *p* = 0.007). Remarkable differences in media consumption are also evident depending on gender: at t0, mean media score for boys is 176 as compared to 129 for girls (95% CI for the difference in means is (24, 70), *p* = 9 × 10^−5^) (Figure 3a) and remained similar at t1 (172 *vs.* 140, (5, 58), *p* = 0.02). No association between (change in) BMI-SDS and media consumption could be found nor was there a significant difference in the mean media score between baseline and end of the intervention.

*Quality of life:* The KINDL-R score is available for 281 participants at t0 and for 194 participants at t1. Between both time points there is minimal difference in the scores between those who provided scores at t1 and those who did not (mean score: 93.7 *vs.* 93.4, *p* = 0.8 for t-test). There is a strong negative linear correlation between quality of life and age in our study cohort, independent of whether questionnaires were filled out by parents (for children < 10 years) or by participants themselves (≥10 years): Correlation coefficient: −0.32, 95% CI (−0.42, −0.21), p = 5 × 10^−8^; Figure 3b). Correcting the KINDL-R score for t1 by using the slope of the regression and the elapsed time between the two questionnaires, a small but significant increase in quality of life by 3.4 points can be found (95% CI (1.7, 5.0)), *p* = 6 × 10^−5^). However, neither a group effect nor an association between KINDL-R score and change in BMI-SDS could be detected.

*Eating patterns:* Data on eating behavior is available for all 289 participants at t0 and for 194 participants at t1. The majority of the participants (claim to) have very good eating habits at the beginning of the intervention (eating pattern score of at least 5 points: 73% of participants Table 1). However, the score decreases with age for participants ≥ 10 years (regression coefficient −0.39 (95% CI (−0.69, −0.09)), *p* = 0.01). No association was found between eating pattern score and (change in) BMI-SDS nor was a difference seen between the groups.

*Physical activity:* Questionnaires on physical activity are available for 287 participants at t0 and for 193 subjects at t1. There is a clear age dependence of physical activity levels for the older children, with a decrease starting approximately at age of 10 (coefficient −1.5 (95% CI (−2.0, −1.0), *p* = 9 × 10^−9^).

## 4. Discussion

We have evaluated the efficacy of a randomized controlled obesity and weight gain prevention study for families with overweight or obese children, based on one-year telephone counseling. Such approaches are necessary, since health systems are faced with the task of providing economically viable weight-loss strategies to a large number of people of various ages, who may be spread over a large area. This study is amongst the first randomized controlled trials analyzing the effect of such an intervention on change in BMI-SDS and a comprehensive set of anthropometric, lifestyle-related and psychosocial data in childhood obesity.

Regarding our main study outcome, we show that differences in success rate and change in BMI-SDS between the intervention- and the control group are small when considering the Full Analysis Set (FAS). However, this analysis follows a true “intention-to-treat” approach and considers data from all randomized participants, including those who dropped out at any time point during the intervention, did not provide data within the defined time frame or did not follow the intervention according to the manual. In the Per Protocol Set (PPS), there is a significantly higher success rate and larger changes in BMI-SDS in the intervention group compared to the control group. This suggests that children were significantly more likely to complete the intervention successfully when they followed the protocol to completion. Those who complete the therapy and follow it according to the protocol tend to be those with more will-power, motivation and perseverance, who may well be better at losing weight [29]. Thus, the PPS approach may not provide a realistic picture of the effect of any given therapy, since excluding data from those participants who did not complete the intervention may induce some bias by producing overly positive results. To date, most research groups essentially use a PPS approach, calling it intention-to-treat, since patients are excluded from the primary analysis if they do not provide data at follow-up.

As with other trials looking at weight loss amongst children and adolescents, we have chosen changes in BMI-SDS as the primary endpoint. The most difficult issue to deal with adequately is that of “informative missings”, *i.e.*, missing data that introduces a bias. In the context of weight loss programs this arises since many of those who terminate the trial without providing data are likely to have failed to lose weight. Thus, the data available from the intervention arm are likely to paint a rosier picture than access to the full data would have yielded. One way of dealing with this problem is to define “success” by some criterion and base results only on positive proof of having been successful while retaining all randomized participants. We have chosen this approach and suggest it as the gold standard when analyzing the efficacy and outcome of obesity intervention trials.

A similar study has investigated the impact of low-intensity, behavioral one-year family lifestyle intervention in 151 participants aged 13–16 years [30]. This study reported a dropout rate of 29% (compared to 31% in our intervention arm) and a significant reduction of BMI-SDS and additional anthropometric and clinical parameters using the PPS approach [30]. Another study has investigated the impact of a family lifestyle intervention in 248 children aged 8–14 years. A drop-out rate of 44% was observed at 9 months and a significant BMI-SDS reduction was seen after one year in the “completers”, *i.e.*, in children who completed the study [31] Although our intervention is comparable, collection of height and weight data during visits to their pediatricians proved to be a problem.

With regard to secondary endpoints, baseline data show that eating patterns of our sample worsened with age for participants older than 10 years. This could suggest an increasing independence from parental eating patterns for children approaching pubertal age. With advancing puberty, parents see their children gaining independence, and the influence of peers on what their offspring eat becomes increasingly important [32].

Regarding physical activity, decreasing activity levels starting at approximately age 10 were observed. This finding is consistent with previous studies, which have shown that the level of physical activity seems to decline during childhood and adolescence, starting at school age, with children spending approximately 10% less time in physical activity for each advancing year of age [33,34]. In addition, the transition time between childhood and adolescence and between primary school and secondary school around the age of 10 years has been previously reported to be a critical age for decrease in physical activity levels [35].

Evaluating media consumption revealed that about half of the candidates spent more than two hours and about one quarter more than 3 h daily consuming some kind of media. We also found a significant dependence on age, starting at about 10 years, as well as a dependence on gender, with girls spending significantly less media time than boys, regardless of age group. Cluster analyses among a representative sample of adolescents in Germany have shown that adolescents with high levels of media consumption are more likely to be overweight [36]. 

Regarding quality of life, we show a strong negative correlation with age, independent of whether questionnaires were filled out by parents or by participants themselves. This finding is in accordance with a very recent study that reported an association between overweight and obesity in adolescents and poorer quality of life when overweight continued over the track of childhood to adolescence [37]. A validation analysis of KINDL-R scores among a representative group of children and adolescents from Germany, regardless of weight status, also documented a decrease of reported quality of life with increasing age, albeit moderate [38]. However, in contrast to our findings, another German study found gender-specific improvements of quality of life, measured with the same instrument (KINDL-R questionnaire), in moderately overweight children and adolescents after outpatient training [39].

In general, the intervention showed no significant change in secondary parameters assessed by self-reported data. Similar results have been reported by other groups, with no or few effects on secondary outcomes such as level of physical activity, eating patterns, psychological variables or sedentary behavior in obesity intervention programs despite some effect on weight status as a primary outcome [30,40]. These results show that the association between weight status and lifestyle habits in childhood obesity is not simple and should be the focus of further research.

Participation rates for obesity interventions are in general rather low, and it remains a challenge to attract obese children and convince them to participate [41,42]. Thus, it is crucial to consider a variety of factors during different stages of treatment when planning to establish and implement the “optimal” intervention program [31,43]. Since motivation of participants seems to be one of the key-players for the success of any intervention, tailored interventions are strongly warranted [31].

An additional aspect to successfully intervene in childhood obesity is the involvement of political stakeholders and governments to minimize the future burden on society from the consequences of childhood obesity. One possible preventive strategy to address the serious public health concern of childhood obesity is taxation of food and drinks, especially sugar-sweetened drinks, as recently suggested in Australia and other countries [44]. In addition, the availability of electronic devices and transport from school to home is associated with weight status in children [45].

Successful childhood obesity prevention strategies are scarce. A recent review has evaluated after-school obesity prevention programs, ranging from kindergarten to middle school aged children. The duration of interventions greatly varies, but most are short-term. As evaluation is often lacking or incomplete, and the interpretation of results is limited [46]. Thus, randomized, controlled trials for prevention of childhood obesity are urgently needed and should involve parents and the rest of the family, as both parenting styles and lifestyle habits are important factors [47,48].

Strength of the current study is that it is amongst the first randomized controlled trials to evaluate the efficacy of a one-year telephone-based obesity prevention program targeted to parents of affected children. Our study included a wide age span of childhood development (3.5–17.4 years) and addressed more age groups than comparable studies. As the comprehensive data collection (anthropometric and clinical data, lifestyle, eating patterns, physical activity behavior, media consumption and quality of life) was performed in mother-father-child triads, detailed analyses of several aspects of childhood obesity and lifestyle choices can provide further insight for the implementation of future studies. However, there are also limitations: As questionnaires were completed by parents (mothers and fathers separately) as well as children older than 10 years themselves, discrete and independent completion of the study material has to be assumed, but could not be controlled for. Since the participants were not seen face to face, it was not always easy to encourage them to be weighed and measured and to return study material at the appropriate time. However, the effect of lag-times was analyzed and not found to have a significant impact on the results.

## 5. Conclusions

A telephone-based obesity prevention program is effective for those who follow to the protocol and to completion. However, these individuals are presumably those with more motivation, will-power and perseverance, who may well be better at losing weight. An honest assessment of such programs shows that the high drop-out rates mean that average weight-loss and success rate are not satisfactory. Eating patterns, leisure time habits and quality of life worsened with age in overweight or obese children and adolescents, independent of the intervention, weight status and weight change. This suggests that the connection between lifestyle and weight status is not at all simple to target and needs to be addressed in future research including intervention programs.

## Supplementary Files

Supplementary File 1
