# Supplementary material for: Telephone-Based Adiposity Prevention for Families with Overweight Children (T.A.F.F.-Study): One Year Outcome of a Randomized, Controlled Trial
Source: Int J Environ Res Public Health. 2014 Oct 3;11(10):10327–44. doi: 10.3390/ijerph111010327 (PMC4210981; doi:10.3390/ijerph111010327)
Supplement: Supplementary File 1 [file ijerph-11-10327-s001.pdf]

Supplementary Information

Telephone-Based Adiposity Prevention for Families with Overweight Children (T.A.F.F.-Study): One Year Outcome of a Randomized, Controlled Trial

---

Supplementary File 1: Rules for Choosing Valid Analysis Parameters

The following rules for choosing valid parameters were applied: For baseline measurements, anthropometric data from the screening process were utilized, and additional parameters were obtained from baseline questionnaires from participants older than 10 years, mothers and fathers. For parameters after termination of the intervention, the closest anthropometric measurement to the final counseling interview was utilized that had to be within the time frame of not more than 90 days before it or 30 after it (intervention group). If the final interview did not take place, the closest anthropometric measurement to completion of the 1-year questionnaires was applied that had to be within the time frame of not more than 90 days before it or 30 after it. As for the control group, the closest anthropometric measurement to the completion of questionnaires at t1 was utilized that had to be within the time frame of not more than 90 days before it or 30 after it. The cut-off of 30 days was chosen during periods of intervention (the control group began with intervention after completing the questionnaire at t1), when weight change can expected to be more rapid, otherwise 90 days was chosen. If there was no final telephone interview (intervention group) nor was the questionnaire filled out at t1 (both groups), the average time lapse between the questionnaires was added to the date of the first questionnaire and the closest measurement not more than 180 days before it or 180 after it was applied for further analyses.

Supplementary File 2: Questions that Formed the Basis for Scores

Supplementary File 2a: Eating Pattern Score

The following questions were included in the eating pattern scale: (i) Number of meals: The question asked was "Which meals do you take regularly during the week and on the weekend?" Possible answers included breakfast, morning snack, lunch, midday snack, supper, with separate questions for the week and the weekend, respectively. Each answer corresponded to one point, taking into account different weighting for weekdays and weekend days. Values between 2–5 counted as negative, 6–7 as neutral and 7–10 as positive; (ii) Eating with the family: The question "Which meals does the whole family eat together?" was applied. Possible answers included breakfast, lunch, supper, no joint meals, which could be selected for the week and the weekend separately. At least one meal during the week and at least two on the weekend were counted as good. "Good" both during the week and on the weekend led to a positive subscore, just one 'good' to a neutral one and two "bads" to a negative one; (iii) Activities while eating: The question was "What do you do while eating and how often"? A list of seven categories was provided, including watch TV, talk with my family, play (computer, mobile), read/write (homework), listen to music, walk around, I don't do

anything else. Four possibilities for describing how often each activity is performed were provided: never/seldom, sometimes, often and daily/frequently, depending on the questionnaire applied. These options were assigned the values 0 (never), 1 (seldom), 2 (sometimes), 3 (often), 4 (frequently) and 5 (daily). A score was determined resulting in values between −10 and 10. Values <−1 are defined to be a negative, between −1 and 6 to be neutral, and above 6 positive; (iv) Regular mealtime subscale: The question "Do you have regular eating hours?" was applied. This question was answered for the week and the weekend separately with the possibilities: no; everyone eats when he/she is hungry; no, the h fluctuate quite a bit but we all eat together; yes, for all the main meals; yes, for at least one meal. Both "no" questions were assigned a value of zero, "for at least one meal" a value of one and "for all the main meals" a value of two.

The values for the week and the weekend were added together and the negative score was assigned to the combined values zero and one, a neutral score to the value two and a positive one to the values three or four. If either week or weekend was missing, then the available value was doubled.

Supplementary File 2b: Physical Activity Score

The following two questions were included in the physical activity score: "How often are you physically active in your free time outside of organized sports (e.g., swimming, playing with friends outside, going to the soccer field/streetball court/playground)"? Possible answers included never/seldom, roughly 1–2 per month, roughly 1–2 per week, roughly 3–5 per week, roughly every day. The second question was "Are you involved in organized sports/at a gym"? Possible answers were no, yes for xxx years, yes for xxx months.

Points were given for both questions and added to obtain the final activity score (ranging between 0.5 and 34). If one of the two questions was unanswered, the lowest value for that question was taken (*i.e.*, 0.5 for the first or 0 for the second question). If both questions were unanswered, it was treated as missing value.

Supplementary File 2c: Media Score

Questions applied for the media score were "How much time do you spend each day playing with the computer/video games/internet/cell phone?" and the second was "How much time do you spend each day watching television/videos/DVDs?" Answers were provided separately for the week and the weekend and the possible choices were never (0 points), rarely (10 points), 30 min daily (30 points), 1–2 h daily (90 points), 3–4 h daily (210 points), ≥5 h daily (300 points). Individual scores were determined by multiplying the number of points for the week by 5/7 and adding it to the number from the weekend multiplied by 2/7. If either week or weekend was missing, the available number of points was used (without a multiplication factor). The scores resulting from both questions were added. If one was missing, then the other was treated as zero.

Supplementary File 2d: Health Related Quality of Life

The KINDL questionnaire consists of 6 categories with four questions each (total of 24 questions). The categories are body/physical well-being, psyche/emotional well-being, self-esteem, family

environment, interaction with friends, school/everyday functioning. Each question can be answered with never, seldom, sometimes, often, always. These are numbered from 1–5 or 5–1 such that 5 is always the most positive answer. The sum of these individual scores yields the final score, ranging from 24 (negative) to 120 (positive). If n questions are unanswered, the sum is multiplied by the factor $24/(24 - n)$. A score is considered missing if more than 7 questions went unanswered.

© 2014 by the authors; licensee MDPI, Basel, Switzerland. This article is an open access article distributed under the terms and conditions of the Creative Commons Attribution license (http://creativecommons.org/licenses/by/4.0/).
